# Supplementary material for: Venomix: a simple bioinformatic pipeline for identifying and characterizing toxin gene candidates from transcriptomic data
Source: PeerJ. 2018 Jul 31;6:e5361. doi: 10.7717/peerj.5361 (PMC6074769; doi:10.7717/peerj.5361)
Supplement: Supplemental Information 3 [file peerj-06-5361-s003.gz › FinalOutput_E-20/Hyaluronidase_conohyal-Cn1_1/finaltree.pdf]

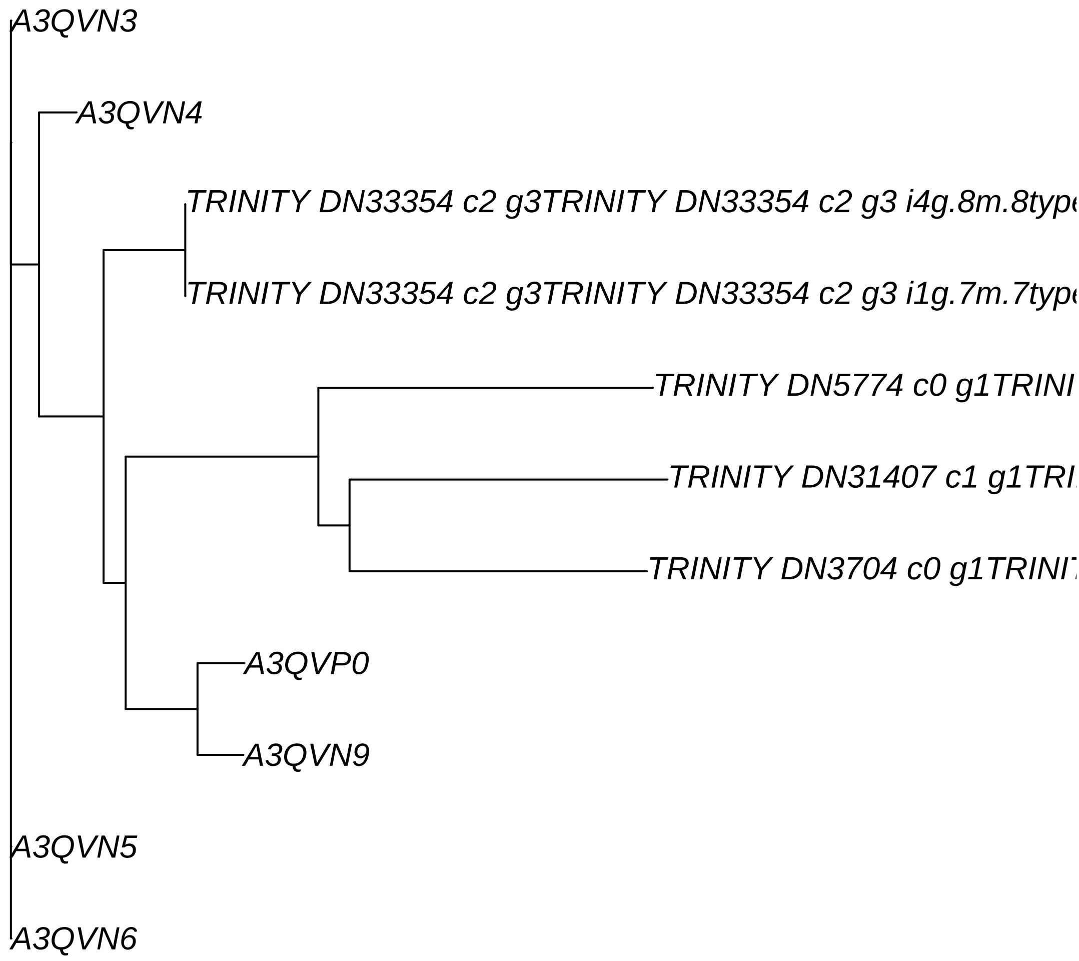
A3QVN3
A3QVN4
TRINITY DN33354 c2 g3TRINITY DN33354 c2 g3 i4g.8m.8type
TRINITY DN33354 c2 g3TRINITY DN33354 c2 g3 i1g.7m.7type
TRINITY DN5774 c0 g1TRINI
TRINITY DN31407 c1 g1TRI
TRINITY DN3704 c0 g1TRINIT
A3QVP0
A3QVN9
A3QVN5
A3QVN6
